# Successful arm replantation using traditional surgical techniques: a case report from a low-resource country

**DOI:** 10.1097/MS9.0000000000002758

**Published:** 2025-01-09

**Authors:** Malaka Abubakir, Lana Sabbagh, Ghaiyath Khalil, Shahama Al Ali, Salim Tfankji, Hussein Alkanj

**Affiliations:** a Faculty of Medicine, University of Aleppo, Aleppo, Syrian Arab Republic; b Department of Vascular Surgery, Aleppo University Hospital, University of Aleppo, Aleppo, Syrian Arab Republic.; c Department of Cardiovascular Surgery, Aleppo University Hospital, University of Aleppo, Aleppo, Syrian Arab Republic

**Keywords:** amputated arm, case report, replantation, traditional techniques, upper limb

## Abstract

**Introduction:**

Upper limb replantation requires rapid intervention by a multidisciplinary team and appropriate equipment to rescue the limb. Note that the success of the operation and the decision to perform it depend on several factors. This case report presents an upper limb replantation performed using traditional surgical techniques.

**Case presentation:**

A 12-year-old boy presented to our hospital with a crushing injury to the right upper arm, which was amputated at the lower third of the humerus, with the biceps brachii tendon remaining intact. The first aid included resuscitation and intravenous fluid administration to maintain hemodynamic stability. The surgeons replanted the arm by immobilizing the fracture with Kirschner wires, performing several arterial and venous anastomoses, and suturing the damaged nerve and muscle aponeurosis in a 3-h operation. The patient was discharged after 5 days without any complications but was recommended to undergo physical therapy to regain his arm movement and is currently undergoing monthly follow-ups to ensure the required improvement.

**Discussion:**

Upper limb replantation is a complicated process that enables the conservation of arm function and appearance. This requires rapid intervention by a multidisciplinary team. Additionally, advanced equipment, such as microsurgical tools, provides greater success and ease of revascularization.

**Conclusion:**

To successfully re-implant an amputated limb, several factors must be considered: surgeon experience, availability of necessary equipment, and provision of post-operative care. Despite lacking advanced tools, surgeons can rely on their surgical skills to overcome this challenge.

## Introduction

Replanting an upper limb is a complex process that requires immediate and rapid intervention from a multidisciplinary team in addition to the availability of appropriate equipment[1]. The first clinical replantation of a limb was performed by Malt and McKhann in 1962[2]. Although upper limb amputation is not usually a life-threatening condition, it negatively affects the overall quality of life of patients[3].

The main aim of traumatic replantation is to achieve optimal functionality[2]. This outcome depends on factors within our control, such as the duration of ischemia, as well as those beyond our control, including age, degree of nerve injury, and the level of amputation[2]. The decision to proceed with limb replantation is based on several factors, including the importance of the organ, level of injury, mechanism of injury, and desired functional outcome[3]. The success of the operation depends on several factors, such as a short duration of ischemia, proper management of the amputated part, type of cutting tool used, and mechanism of injury[4].

Unfortunately, owing to the harsh circumstances resulting from the war, there is a notable absence of advanced equipment and tools. Consequently, surgeons faced a major challenge in saving the limb of a 12-year-old child who was admitted to the hospital after experiencing a crushing injury to the arm, which damaged all structures except the biceps brachii tendon and the surrounding skin. This case report has been reported in line with the Surgical CAse REport “SCARE” 2023 Criteria[5].

## Case presentation

**Figure 1.**
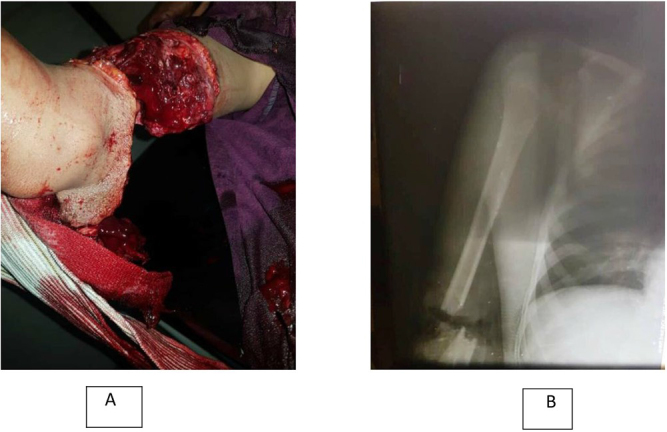
(A) The amputated part upon arrival, the arm was subtotally amputated at the lower third of the humerus, with the biceps brachii tendon remaining intact. (B) Preoperative X-ray.

A 12-year-old boy presented to the emergency department with a crushing injury caused by a kneading machine. The right upper arm was amputated in the lower third of the humerus, with the biceps brachii tendon remaining intact (Fig. 1A).

On arrival, the warm ischemia time was 4 h with Glasco coma scale 13. The limb was tied with a cloth to prevent bleeding and presented pallor and coolness. Initial intervention in the emergency room included resuscitating the patient following The Air way, Breathing, Circulation, Disability, Exposure “ABCD” approach and administering fluid replacement with intravenous fluids to maintain hemodynamic stability. An arm X-ray revealed a displaced fracture of the lower humerus with a change in the bone’s axis (Fig. 1B). Physical examination revealed material loss in the medial and posterior sections of the lower third of the humerus, along with bone crepitus. The pulse was unpalpable in the brachial artery at the elbow, as well as in the ulnar and radial arteries, with no signal appearing on arterial Doppler ultrasound examination. Moreover, the median nerve was cut, also the ulnar and the radial nerves were transected at the level of the lower humerus. Laboratory tests revealed low hemoglobin levels and a reactive increase in WBCs. The patient’s family confirmed that the patient did not have any significant medical or surgical history prior to the operation.

**Figure 2.**
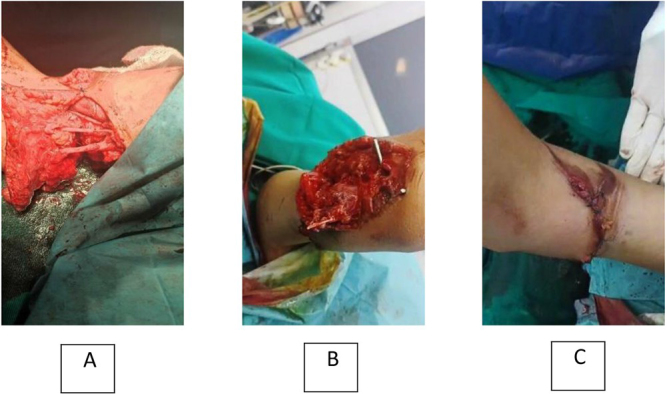
(A) The end-to-end anastomosis of the brachial artery, basal vein, and the ulnar nerve. (B) Material loss of skin in the lateral part of the lower arm. (C) The final result after muscles approximation and skin closure, medial part of the lower humerus.

The surgical team, which consisted of vascular, orthopedic, and neurosurgeons, decided to perform a replantation operation, starting with the administration of general anesthesia and povidone-iodine sterilization. The orthopedic surgeon immobilized the distal humerus fracture using two Kirschner wires. Vascular surgeons found out that the brachial artery was cut with clots inside it, so they isolated the distal and proximal sections of the brachial artery and utilized a Fogarty catheter (2F) to restore blood flow, followed by an end-to-end anastomosis with a (7/0) Prolene suture, ensuring pulse restoration. The basilic vein was isolated in its proximal and distal segments, and an end-to-end anastomosis was performed using a (7/0) Prolene suture. The cephalic vein was also isolated, and its integrity was confirmed to ensure proper venous branch homeostasis (Fig. 2A). The total warm ischemia time was approximately 7 h. The neurosurgeon isolated the proximal and distal segments of the ulnar and radial nerves and then sequentially sutured them using (7/0) Prolene sutures (Fig. 2A). Additionally, the median nerve was isolated in its proximal and distal segments and sutured with (7/0) Prolene sutures as well. Finally, vascular surgeons sutured the muscle aponeurosis and closed the wound using sutures and a sterile dressing. The skin was left to heal by the second intention due to significant skin loss, completing the operation within 3 h (Fig. 2B and 2C).

During the operation, the patient received a tetanus vaccine along with ceftriaxone 1 g, 2500 IU of heparin as a loading dose and one unit of blood alongside one unit of plasma.

**Figure 3.**
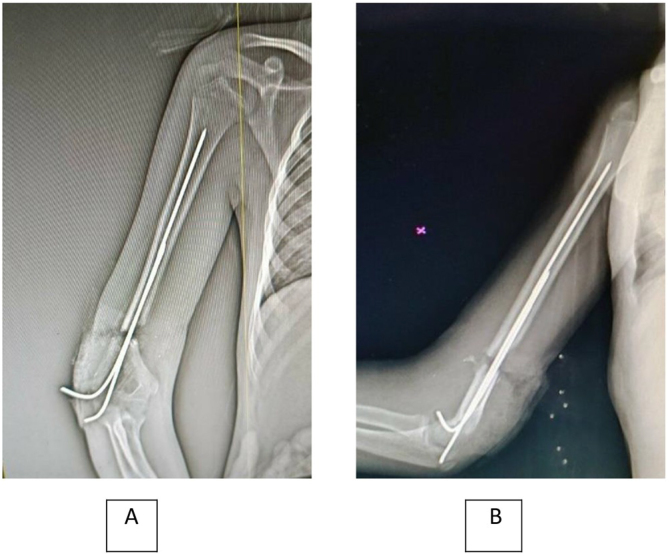
(A) Post-operative anterior X-ray. (B) Post-operative posterior X-ray; showing the internal fixation by k. wires.

After the operation, an X-ray was performed to assess the condition of the arm (Fig. 3A and 3B). The prescribed regimen of medications included ceftriaxone following an allergy test and cetamol, flagyl, amikacin, heparin, and saline serum on a daily basis. The initial outcomes of the operation revealed successful revascularization of the limb, as demonstrated by the restoration of normal capillary refill time and the finger oximeter registering an oxygenation rate of 97%. The limb had healthy coloration.

**Figure 4.**
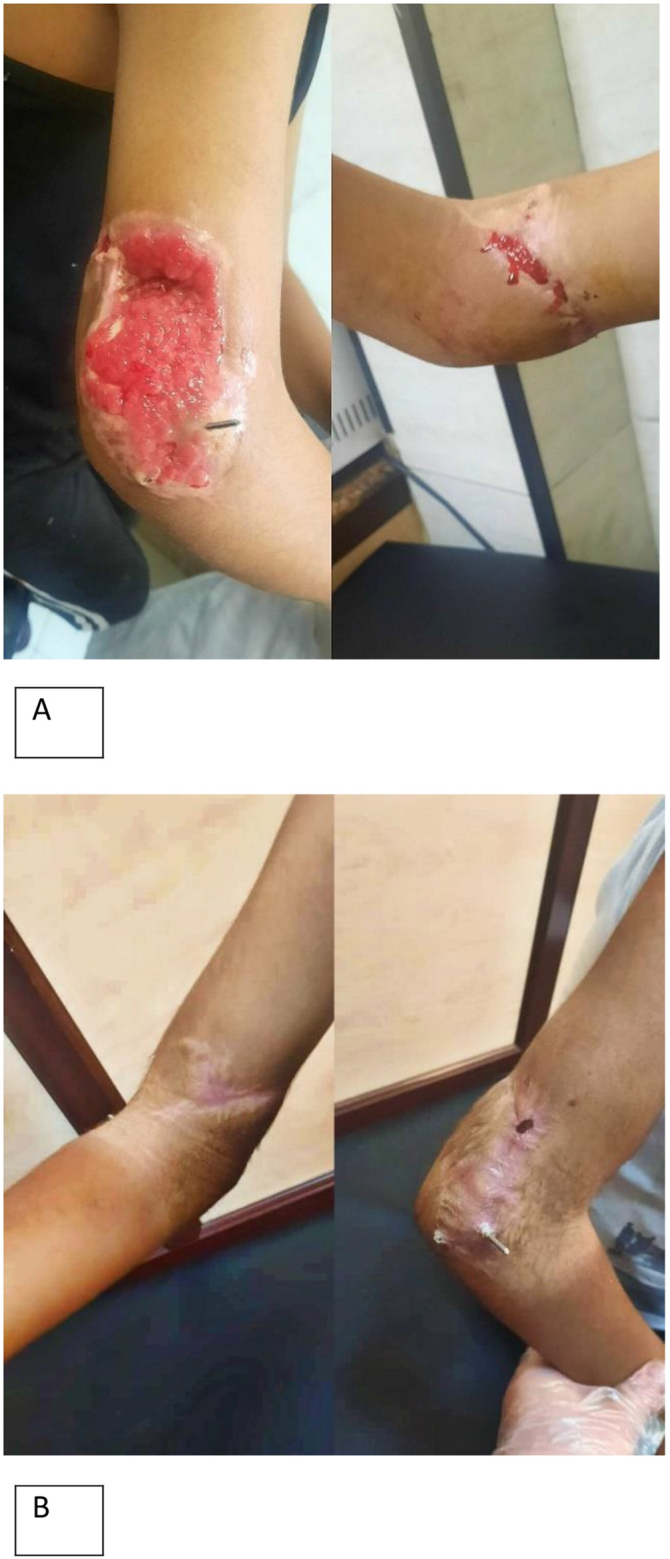
(A) The arm after 2 weeks at the follow-up. (B) The arm after 3 months at the follow-up.

The patient did not need to stay in the intensive care unit. After a post-operative observation period of 5 days, the patient did not experience any complications such as edema or infection, which allowed for discharge. The patient underwent weekly follow-up visits, and after 3 months, a palpable pulse was observed. Wound rehabilitation demonstrated excellent improvement (Fig. 4A and 4B); however, limb movement remained impaired, with associated paresthesia along the ulnar nerve distribution. The cosmetic appearance was satisfactory with anticipated potential residual scarring. Patient satisfaction was high owing to successful limb preservation.

Recommendations for the patient included initiating physical therapy to enhance limb mobility and scheduling an electromyography evaluation after 6 months, with ongoing weekly follow-up.

## Discussion

Upper limb replantation presents a significant challenge for surgeons, primarily aimed at conserving both the function and aesthetics of the arm[1]. This complex procedure requires a multidisciplinary team that includes vascular, neurological, and orthopedic surgeons[1]. Patients with traumatic amputation injuries are managed in the emergency department according to the Advanced Trauma Life Support Protocol[6]. The initial management steps before the replantation procedure involve controlling bleeding, administering intravenous fluids to prevent volume loss, keeping the patient warm to avoid hypothermia, and providing prophylactic tetanus and antibiotics^[3,7]^. The amputated part should be immersed in saline-moistened gauze rather than in water and placed inside a plastic bag. Then, the plastic bag should be placed on ice^[3,8]^. It is crucial to not place the amputated part directly on ice, as this can lead to vasospasm, compromising the residual vascular supply^[3,8]^. Intraoperatively, surgeons should first identify and safeguard the nerves, blood vessels, and tendons; shorten the bone to alleviate tension on the neurovascular bundle; employ K-wire fixation; ensure adequate arterial blood flow; and repair the arteries and veins through anastomosis. If feasible, all tendons should also be repaired[9].

Post-operatively, anticoagulation therapy is typically recommended[3]. The patient’s room should be kept warm, and the affected limb should be positioned at the heart level to reduce edema without hindering blood flow[3]. The success of replantation is greatly influenced by the level and mechanism of injury, as well as the surgeon’s experience[10]. Moreover, the Chen classification was developed for the post-operative assessment of upper extremity replantation; this classification is preferred because it effectively evaluates the success of replantation across various levels of amputation[3]. It assesses the ability to return to work, perform daily activities, recover sensations, and achieve a range of motion in the affected joint[3].

Several post-operative complications may arise, including early complications due to infection or microvascular breakdown[10]. Late complications may include cold intolerance, joint ankylosis, neuropathic pain, tendon adhesions, bone malunion, and nonunion[10]. Physiotherapy and occupational therapy are crucial in assisting the patient to recover as much function as possible[9]. Technical advances in microsurgery with modern tools, such as microscopes and microsurgical instruments, have significantly increased the success rate of revascularization after limb amputations^[3,11]^. However, due to the circumstances in Syria and the limited resources available, we had to utilize traditional surgical techniques to repair the affected structures.

## Conclusion

To conclude, upper limb replantation is a serious surgical procedure that requires a multidisciplinary team and a specialized center equipped with essential equipment, such as microscopes and microsurgical instruments. This case describes the successful replantation of an amputated arm in a low-resource country, achieved despite the absence of these essential equipment, a satisfactory result, and saved a child’s arm, owing to our team’s surgical expertise using traditional surgical techniques. The patient is currently undergoing physical therapy and monthly follow-up to ensure proper functional restoration.

## Data Availability

The datasets used during the current study are available from the corresponding author upon request.
